# Off-target effect of the BMI1 inhibitor PTC596 drives epithelial-mesenchymal transition in glioblastoma multiforme

**DOI:** 10.1038/s41698-019-0106-1

**Published:** 2020-01-06

**Authors:** Anthony Flamier, Mohamed Abdouh, Rimi Hamam, Andrea Barabino, Niraj Patel, Andy Gao, Roy Hanna, Gilbert Bernier

**Affiliations:** 1 Stem Cell and Developmental Biology Laboratory, Hôpital Maisonneuve-Rosemont, 5415 Boul. l’Assomption, Montréal, H1T 2M4 Canada; 2 Department of Neurosciences, University of Montreal, Montreal, Canada; 3 Present Address: Whitehead Institute of Biomedical Research, 455 Main Street, Cambridge, 02142 MA USA

**Keywords:** CNS cancer, Cancer stem cells, Epigenetics

## Abstract

Glioblastoma multiforme (GBM) is an incurable primary brain tumor containing a sub-population of cancer stem cells (CSCs). Polycomb Repressive Complex (PRC) proteins BMI1 and EZH2 are enriched in CSCs, promoting clonogenic growth and resistance to genotoxic therapies. We report here that when used at appropriate concentrations, pharmaceutical inhibitors of BMI1 could efficiently prevent GBM colony growth and CSC self-renewal in vitro and significantly extend lifespan in terminally ill tumor-bearing mice. Notably, molecular analyses revealed that the commonly used PTC596 molecule targeted both BMI1 and EZH2, possibly providing beneficial therapeutic effects in some contexts. On the other hand, treatment with PTC596 resulted in instant reactivation of EZH2 target genes and induction of a molecular program of epithelial–mesenchymal transition (EMT), possibly explaining the modified phenotype of some PTC596-treated tumors. Treatment with a related but more specific BMI1 inhibitor resulted in tumor regression and maintenance of cell identity. We conclude that inhibition of BMI1 alone is efficient at inducing GBM regression, and that dual inhibition of BMI1 and EZH2 using PTC596 may be also beneficial but only in specific contexts.

## Introduction

Glioblastoma multiforme (GBM) represents the most common brain malignancy in adults. However, current treatments are mostly effective at reducing intracranial brain pressure and the alkylating agent Temozolide (Temodal) can increase lifespan by ~4 months. The median lifespan of patients at the time of diagnosis is still 9–12 months. An effective treatment is thus critically needed.^1–3^ GBMs are highly heterogeneous tumors containing a relatively rare sub-population of cancer-initiating cells expressing the CD133 (*PROM1*) cell surface antigen.^4–6^ Based on cell culture and xenotransplantation experiments, it was shown that CD133+ cells behave like neural stem cells, express stem cell markers, and are able to generate new brain tumors in serial transplantations.^5–7^ The CD133+ cancer stem cell (CSC) fraction also represents the radio-resistant cell population in GBM and is believed to be responsible for brain tumor reoccurrence after radiotherapy treatments.^8–10^ Importantly, cell lines grown under serum conditions are not representative of the phenotype of primary GBM tumors.^7^

Transcription factors (TFs), such as SOX2 and OLIG2, have been reported as key molecular cues for gliomagenesis and tumor maintenance.^4,11,12^ More specifically, TLX, ZFHX4, and MLL5 are TFs and chromatin remodelers overexpressed in glioma CSC population and important for their self-renewal.^13–15^ Although the interconnection between these factors remains unclear, we can hypothesize that CSC identity relies on a self-sustaining network of TFs. Notably, it has been proposed that the CSC phenotype and other key features of cancer cells may be driven by an epigenetic circuitry in conjuncture with genetic mutations.^16^

Polycomb Repressive Complexes (PRCs) form large multimeric complexes responsible for the remodeling of the chromatin and can promote gene silencing through specific histone modifications.^17^ They are classically subdivided into two groups, namely PRC1 (which includes BMI1, RING1a, and RING1b/RNF2) and PRC2 (which includes EZH2, EED, and SUV12).^18^ The subsequent histone modifications induced by PRC1 and PRC2 complexes are critical to maintain stable silencing of both euchromatin and facultative heterochromatin.^19–21^ The PRC2 is able to silence chromatin through its histone H3 tri-methylase activity at lysine 27 (H3K27^me3^) while the PRC1 uses histone H2A mono-ubiquitin ligase activity at lysine 119 (H2A^ub^).^19–21^ Studies on the course of mouse development showed that H3K27^me3^ deposition by PRC2 is thought to be a pioneer event required for PRC1 recruitment at developmental genes. Conversely, H2A^ub^ mark by PRC1 may be necessary for H3K27^me3^ deposition and maintenance in somatic cells, constituting a positive feedback loop.^22^ Members of the PRC have been identified as proto-oncogenes in human cancers.^23–28^ *BMI1* (B-cell specific Moloney murine leukemia virus integration site 1) is one of those and initially described as an oncogenic partner in lymphomagenesis. *BMI1* has been found to be overexpressed in several cancers and been shown to be crucial for cancer cell survival in medulloblastoma and glioblastoma.^10,29–36^ Consequently, *BMI1* inhibition in human or mouse GBM cells results in impaired CSC self-renewal and absence of tumor formation in grated mice, and this independently of a functional *Ink4a* locus.^29,36^ Intriguingly, *BMI1* overexpression can confer self-renewal properties and is apparently sufficient to “reprogram” mouse astrocytes into neural stem cells or mouse retinal progenitors into retinal “stem cells”.^37,38^

GBM tumors have been classified into three major sub-types based on differential gene expression. The *proneural/neural* subtype enriched for *DLL3*, *OLIG2*, *ASCL1*, *PDGFRA*, *IDH1*, and *PROM1*; the *classical* subtype enriched for *FOXO3*, *NES* (Nestin), *EGFR* and *AKT2*; and the *mesenchymal* subtype enriched for *CD44* (SSEA1), *CHI3L1*, *NF1*, *TIMP1*, and *TGFβ*.^39^ Notably, it was recently suggested that *EZH2* is enriched in the proneural subtype and *BMI1* in the mesenchymal subtype.^40^ Hence, proneural tumors were apparently resistant to BMI1 inhibition using PTC596, and mesenchymal tumors apparently resistant to EZH2 inhibition using two distinct inhibitors. Based on this, it was proposed that dual inhibition of BMI1 and EZH2 is more efficient at eradicating GBM then when using BMI1 or EZH2 inhibitors alone.^40^

Herein, we present evidences that independently of BMI1 expression level or of the GBM subtype, GBM neural spheres are sensitive to the related BMI1 inhibitors PTC596 and A1016 at a range between 5 and 50 nM. We found that in contrast with A1016, PTC596 markedly interfered with EZH2, FOXG1, and SOX2 protein levels. RNA sequencing (RNA-seq) analyses confirmed that A1016 more closely aligned than PTC596 with the *BMI1* knockout gene expression profile, and that PTC596-treated GBM spheres showed activation of an epithelial to mesenchymal transition (EMT) molecular program and de-repression of PRC2-target genes. In terminally ill mice bearing intracranial tumors, treatments with high concentrations of PTC596 significantly extended median and maximal lifespan. In some but not all grafts, however, relapsing PTC596-treated tumors showed reduced BMI1, EZH2, and SOX2 expression, suggesting epigenetic drift. We conclude that inhibition of BMI1 is highly efficient at eliminating GBM tumors and that treatments with PTC596, which targets BMI1 and EZH2, may be only beneficial in specific contexts.

## Results

**Fig. 1:**
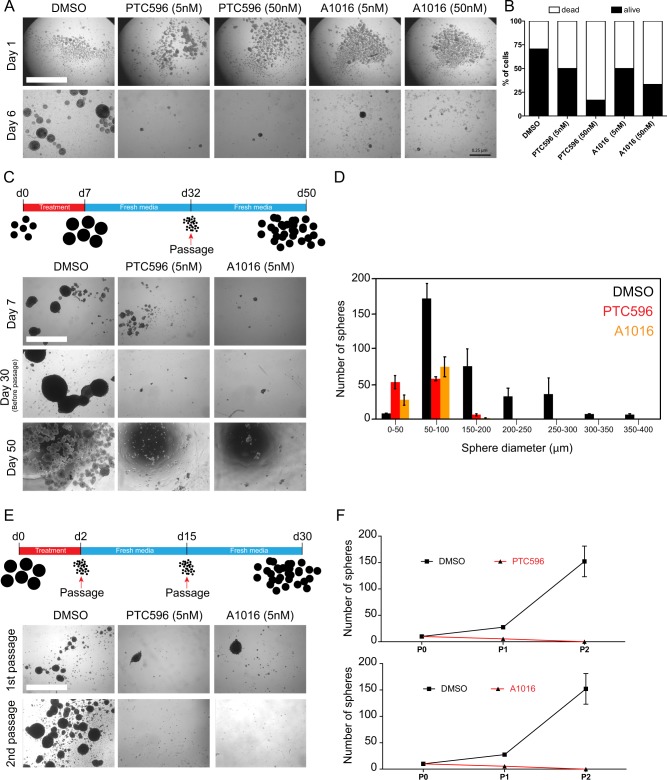
BMI1 inhibitors impair GBM colony growth and cancer stem cell self-renewal. **a** Representative images of GBM0811 cells treated for 6 days by DMSO, PTC596 (5 and 50 nM), and A1016 (5 and 50 nM). Scale bar: 2.5 mm. **b** Quantification of cell viability in GBM0811 cells treated for 6 days by DMSO, PTC596 (5 and 50 nM) and A1016 (5 and 50 nM). **c** GBM1205 cell growth assay upon acute treatment (7 days) with BMI1 inhibitors PTC596 and A1016. Top: scheme of the assay. Bottom: representative images at each time point. Scale bar: 2.5 mm. **d** Size distribution of the spheres at day 50 from experiment in **c** after PTC596 or A1016 treatments in comparison to DMSO-treated cells. **e** Colony-forming assay after acute treatment (2 days) with BMI1 inhibitors and two passages of the GBM0410 cells. Top: scheme of the assay. Bottom: representative images at each time point. Scale bar: 2.5 mm. **f** Quantification of the number of spheres after one and two passages in PTC596 (Top) or A1016 (Bottom) treated cells.

PTC596 is a cell-permeable small compound capable of inducing BMI1 protein proteosomal degradation at nanomolar concentrations.^41^ The compounds can cross the blood–brain barrier and is orally administrable.^40^ To evaluate its utility for brain cancer treatment, we exposed the patient-derived GBM0811 cell line maintained and grown as neurospheres to various concentrations of PTC596, or to A1016, a closely related molecule. After 7 days of treatment, colony growth and cell viability were measured, revealing that both drugs efficiently reduced colony growth starting at 5 nM (Fig. 1a). This also correlated with an important reduction of cell viability in the remaining spheres (Fig. 1b). To evaluate the effect on CSC self-renewal, cancerous neurospheres of the GBM1205 cell line were treated for 7 days with 5 nM of drugs, washed, and maintained for an additional 25 days in drug-free media. Remaining spheres were then dissociated at 4600 viable cells/well in fresh media and maintained for an extra 18 days. We found that both drugs dramatically affected the number and size of newly formed colonies, suggesting depletion of the CSC population (Fig. 1c, d). Likewise, neurospheres of the GBM0410 cell line exposed to 5 nM of drug for 2 days were dissociated in drug-free media (Fig. 1e). After 2 weeks, newly formed neurospheres were re-dissociated in drug-free media to measure secondary sphere formation (Fig. 1e). This revealed that acute exposure of the GBM spheres was sufficient to affect the formation of primary and secondary neurospheres, again suggesting depletion of the CSC population (Fig. 1f).

To test the effect on colony growth, GBM cells were cultured as a monolayer on matrigel in neural stem cell media until large colonies were present. The cultures were then exposed to 5 nM of BMI1 inhibitors for 3 days (Fig. S1A). In all cell lines tested, we observed a severe reduction in colony size and evidences of cell death (Fig. S1A–C). Moreover, we noticed that viable GBM cells rapidly underwent neural (MAP2) and glial (GFAP) differentiation upon exposure to PTC596, as revealed using immunofluorescence (Fig. S1D). Real-time RT-PCR analyses also revealed that *BMI1* mRNA expression was not affected by the drug treatment, but that *PROM1*, a marker of CSC, was decreased in a dose-dependent manner upon treatment with both inhibitors (Fig. S1E). Taken together, these results were consistent with our previous work showing that *BMI1* knockdown resulted in GBM stem cell differentiation and in the loss of self-renewal capacity.^29^

**Fig. 2:**
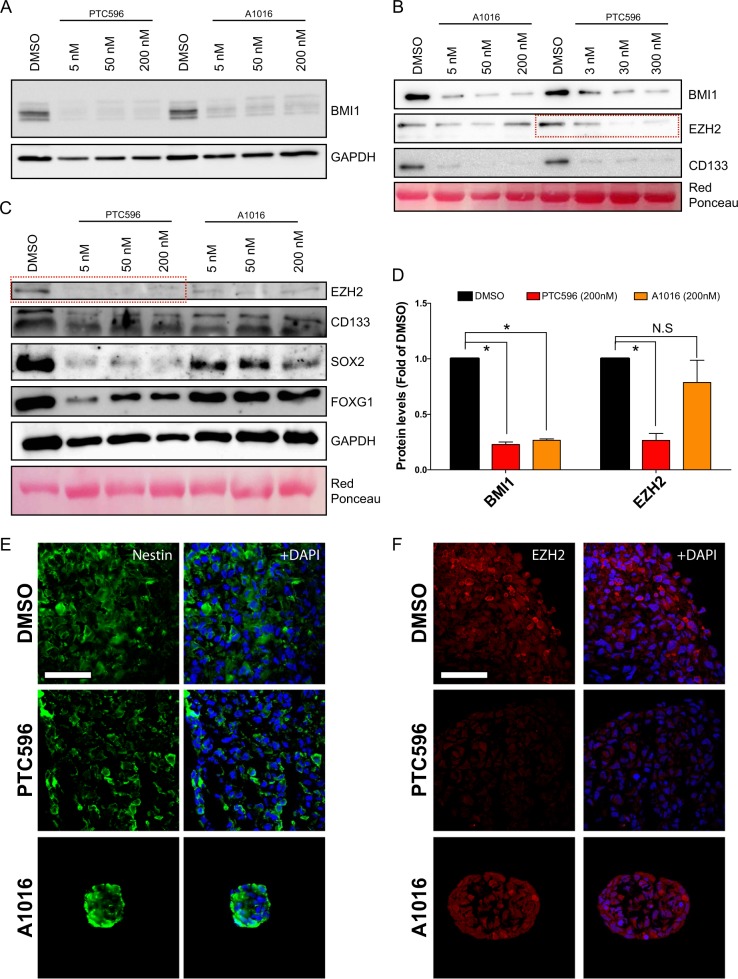
PTC596 inhibits BMI1 and EZH2 in GBM neurospheres. **a** Immunoblot of BMI1 in GBM line 1205 after treatment with increasing concentrations of BMI1 inhibitors. GAPDH is used as a loading control. **b** Immunoblot of BMI1, EZH2, and CD133 in GBM line 0811 after treatment with increasing concentrations of BMI1 inhibitors. Actin and Red ponceau are used as a loading control. Note the reduced EZH2 levels in PTC596-treated cells (dashed red box). **c** Immunoblot of SOX2, EZH2, FOXG1, and CD133 in GBM line 1205 after treatment with increasing concentrations of BMI1 inhibitors. GAPDH and Red ponceau are used as a loading control. Note the reduced EZH2 levels in PTC596-treated cells (dashed red box). **d** Quantification of BMI1 and EZH2 protein levels in GBM cells (*n* = 2 cell lines) after treatment with BMI1 inhibitors (200 nM). All values are mean ± SEM. *P value <0.05. **e** Immunofluorescence for Nestin on GBM line 1205 neurospheres cryo-sectioned after 48 h of treatment with DMSO, PTC596, or A1016. Scale bar: 20 μm. **f** Immunofluorescence for EZH2 on GBM line 1205 neurospheres cryo-sectioned after 48 h of treatment with DMSO, PTC596, or A1016. Scale bar: 20 μm.

**Fig. 3:**
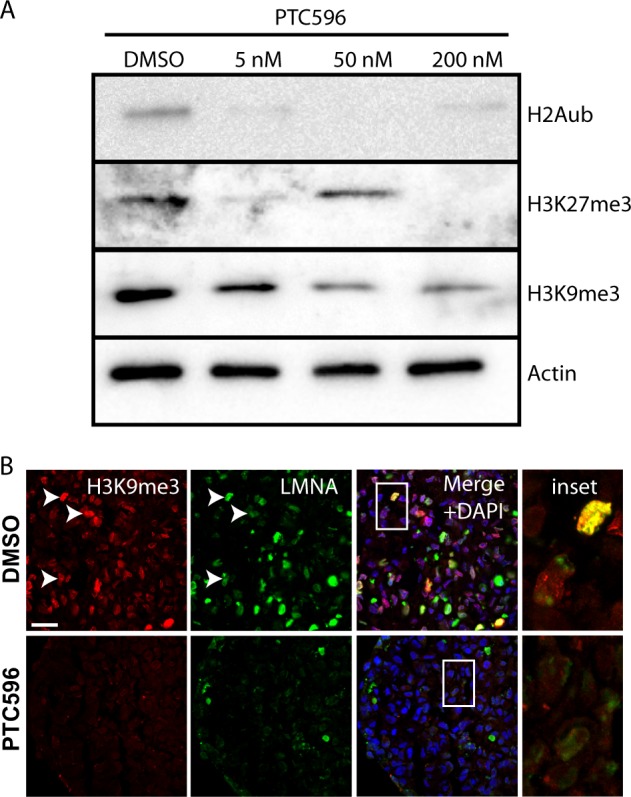
PTC596 treatment perturbs several histone modifications and the integrity of the nuclear lamina. **a** Immunoblot analysis of GBM neurospheres (GBM1205 cell line) treated with PTC596 for 24 h. Note the dose-dependent reduction in H2A^ub^, H3K27^me3^, and H3K9^me3^ levels. **b** Immunofluorescence analysis on sections of GBM neurospheres (GBM1909 cell line) treated with PTC596 for 24 h. Note the reduced H3K9^me3^ land LaminA/C (LMNA) levels in drug-treated GBM spheres. Scale bar: 20 μm.

To study the molecular effects of BMI1 inhibitors, we performed western blot analyses on GBM neurospheres treated for 24 h with increasing concentrations of PTC596 or A1016. DMSO was used as a control. BMI1 inhibition in the GBM0811 and GBM1205 cell lines was observed already at 5 nM, with maximum inhibition reached between 5 and 50 nM, depending on the cell line (Fig. 2a, b). In time course studies, BMI1 inhibition was observed after ~5 h with A1016, and after ~8 h with PTC596 (not shown). To test the effect on the CSC phenotype, we measured CD133, EZH2, FOXG1, and SOX2 protein expression using immunoblot.^29,42–46^ In both cell lines tested, BMI1 inhibition for 24 h resulted in a significant reduction of CD133 (Fig. 2b, c) and H2A^ub^ (Fig. 3a) levels. Surprisingly, we found that the expression of EZH2, FOXG1, and SOX2 was also significantly reduced in PTC596-treated cells, whereas it was almost unchanged in A1016-treated cells (Fig. 2b–d). Using immunofluorescence on GBM sphere sections, we observed that Nestin expression remained unaffected after treatment with PTC596 or A1016 for 24 h (Fig. 2e). However, while EZH2 expression remained in normal ranges in A1016-treated spheres, it was highly reduced in spheres treated with PTC596 (Fig. 2f).

BMI1 is highly expressed in mature human and mouse cortical neurons and its expression is reduced in neurons from Alzheimer’s disease patients.^47,48^ Furthermore, acute BMI1 knockdown in cultured human cortical neurons leads to severe neurodegeneration,^48^ thus raising substantial concerns about possible side effects of BMI1 inhibitors on normal brain function. To investigate this, we generated day in vitro 35 post-mitotic cortical neurons through directed differentiation of human embryonic stem cells or induced pluripotent stem cells.^48^ Cultured neurons were treated with 100 nM of PTC596 or A1016 for 24 h and analyzed by immunoblot. Notably, drug-treated neurons were apparently healthy and presented elevated levels of BMI1 and H2A^ub^, suggesting a concomitant increase in the biochemical activity of the PRC1 (Fig. S2A). Similarly, oral administration of PTC596 or A1016 at 12 mg/kg in post-natal day 45 mice was not accompanied by Bmi1 down-regulation when whole cortices were analyzed by immunoblot (Fig. S2B) or immuno-histochemistry (not shown) 24 h after treatment. To support these observations, stem cell-derived post-mitotic neurons and neural progenitors were exposed to PTC596 or A1016 for 24 h and test for cell viability. While neurons exposed to BMI1 inhibitors were relatively unaffected, neural progenitors showed decreased viability as revealed using the MTT assay or by measuring the proportion of dying cells (Fig. S3A, B). To evaluate the mechanism of action of the new A1016 molecule on BMI1, lysates from neural progenitors exposed to 100 nM of A1016 for 24 h were immuno-precipitated with an antibody against poly-ubiquitin at lysine 48, a mark for protein degradation by the proteasome. This revealed increased poly-ubiquitinated BMI1 in A1016-treated cells, as measured using western blot analysis (Fig. S3C). These results suggested that the inhibitory activity of PTC596 and A1016 on BMI1 is cell cycle dependent and thus less likely to affect the viability of post-mitotic neurons.

EZH2 is the catalytic sub-unit of the PRC2 and is required for H3K27^*me*3^ deposition and maintenance.^49^ On the other hand, the BMI1//RING1 complex regulates H2A^ub^ deposition, possibly also directly or indirectly affecting H3K9^me3^ levels in normal somatic cells.^50^ Consistently, we found that treatment of GBM spheres (GBM1205) with PTC596 for 24 h resulted in robust down-regulation of H2A^ub^ levels (Fig. 3a). Notably, this was also accompanied by down-regulation of H3K27^*me*3^ and H3K9^me3^ levels (Fig. 3a). The heterochromatin is tightly associated with the nuclear envelope and loss of H3K9^me3^ can result in nuclear lamina disintegration.^51^ Using immunofluorescence, we observed that GBM spheres (GBM1909) treated for 24 h with PTC596 showed reduced H3K9^me3^ levels and disintegration of the nuclear lamina (Fig. 3b). This revealed that PTC596 treatment in GBM cells significantly perturbed PRC1- and PRC2-mediated histone modifications and structure of the nuclear envelope.

**Fig. 4:**
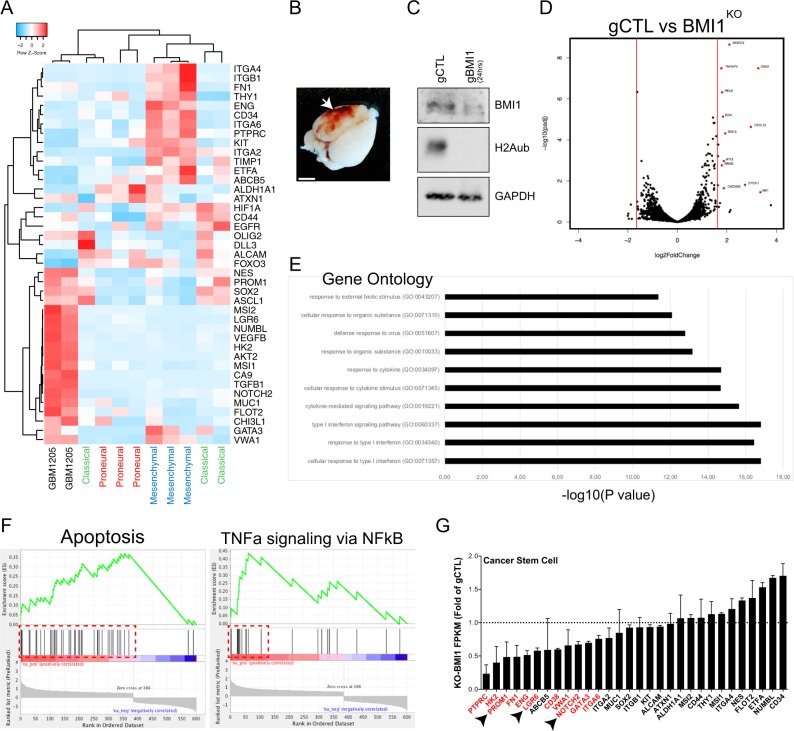
Acute *BMI1* knockout in GBM induces an inflammation-related response and down-regulation of cancer stem cell genes. **a** Heatmap of gene expression for glioblastoma genes in GBM line 1205 (*n* = 2) and GBM tumors having either a classical (green), mesenchymal (blue), or proneural (red) phenotype (*n* = 3 for each group). Row *z*-scores are a function of FPKM value. **b** Image of a NOD/SCID mice brain after intracranial injection of the GBM line 1205. Scale bar: 4 mm. **c** Immunoblot of BMI1 and H2Aub in GBM line 1205 transfected with a non-template control guide RNA (gCTL) or a guide RNA targeting BMI1 (gBMI1) with a plasmid overexpressing Caspase 9. GAPDH is used as a loading control. **d** Volcano plot showing the most dysregulated genes in BMI1^KO^ versus gCTL GBM line 1205. Red dots show significantly dysregulated genes. **e** Selection of Gene Ontology terms for upregulated genes in BMI1^KO^ versus gCTL GBM line 1205. **f** Gene Set Enrichment Analysis (GSEA) showing enrichment for apoptosis and TNFa gene sets in BMI1^KO^ versus gCTL GBM line 1205. **g** Gene expression levels of cancer stem cells genes in BMI1^KO^ versus gCTL GBM line 1205. Red annotations show significantly downregulated genes.

Next, we further analyzed the novel patient-derived cell line, GBM1205, using RNA-seq. When compared to primary GBM tumors from the IVY GAP repository, we determined that the GBM1205 cell line had a mixed phenotype, showing a proneural phenotype with some classical features (Fig. 4a). The cell line was also able to generate hemorrhagic brain tumor in grafted immune-deficient mice (Fig. 4b). To dissect the pioneer molecular changes resulting from *BMI1* inactivation, we targeted *BMI1* exon 1 using CRISPR/Cas9 technology to generate *BMI1* knockout (*BMI1*^*KO*^) GBM cells.^48^ A non-targeting template guide RNA was used as a negative control. Control (*BMI1*^*+/+*^) and *BMI1*^*KO*^ cells were collected 24 h post-transfection for western blot and RNA-seq analyses. We estimated that ~70% of the cells were targeted, and *BMI1*^*KO*^ cell extracts exhibited reduced BMI1 and H2A^ub^ levels, confirming *BMI1* deletion (Fig. 4c). Differential expression analysis revealed that most de-regulated genes in *BMI1*^*KO*^ cells were upregulated. This is consistent with BMI1, working within the PRC1, functioning as a gene-silencing factor (Fig. 4d).^21,52,53^ Gene ontology (GO) annotation of the most upregulated genes in *BMI1*^*KO*^ showed significant appearance of interferon, cell metabolism, chromatin remodeling, and apoptosis-related GO terms (Figs. 4e and S4). Using Gene Set Enrichment Analysis (GSEA), we found an enrichment for apoptosis gene set in *BMI1*^*KO*^, but gene expression levels remained relatively low (Fig. 4f). Notably, *BMI1*^*KO*^ GBM cells were also enriched in genes regulated by NF-κB in response to TNFα signaling, suggesting an inflammation-related cellular response (Fig. 4f). Notably, the CSC gene expression profile was also altered upon *BMI1* inactivation, including that of *PROM1*. Hence, the expression level of 14 stem cell-related genes, all associated with a proneural GBM phenotype, was significantly reduced in *BMI1*^*KO*^ cells, suggesting that BMI1 may be involved in the maintenance of the proneural CSC phenotype in GBM (Figs. 4g and S5).^54,55^

**Fig. 5:**
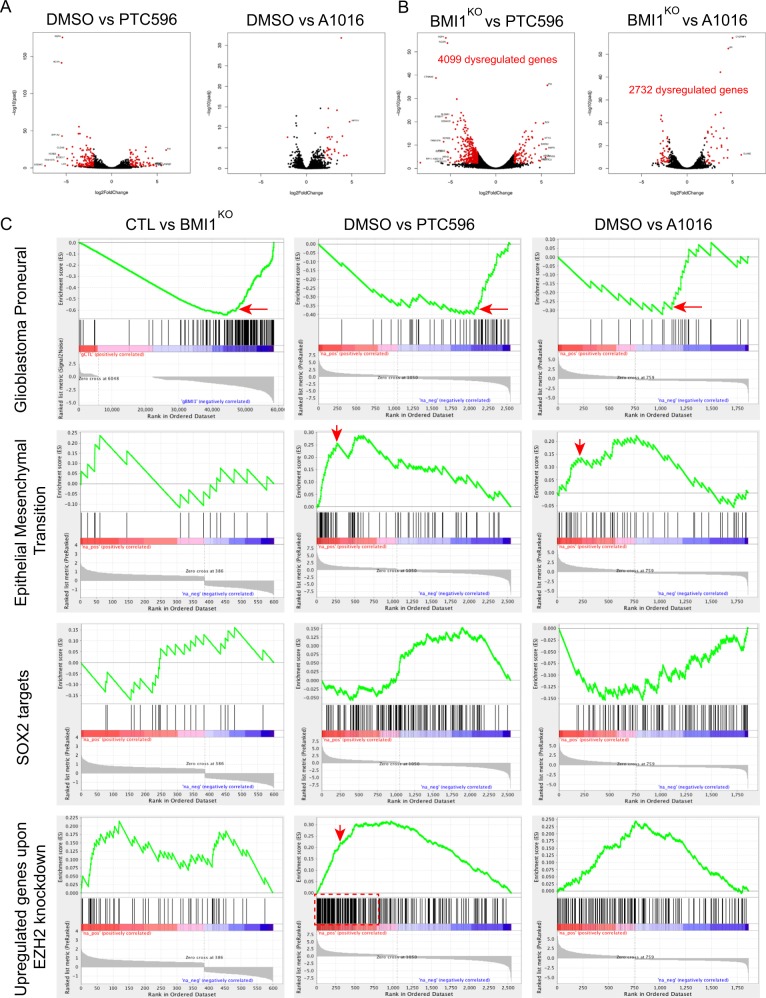
PTC596 perturbs the expression of EZH2 and SOX2 target genes. **a** Volcano plot showing the most dysregulated genes in DMSO versus PTC596- or A1016-treated GBM line 1205 for 24 h. Red dots show significantly dysregulated genes. **b** Volcano plot showing the most dysregulated genes in BMI1^KO^ versus PTC596- or A1016-treated GBM line 1205 for 24 h. Red dots show significantly dysregulated genes. **c** Gene Set Enrichment Analysis (GSEA) showing enrichment for glioblastoma proneural, epithelial–mesenchymal transition, SOX2 targets, and EZH2 targets gene sets in BMI1^KO^, PTC596, or A1016 GBM line 120516. Red arrows and boxes highlight enrichments of interest.

To assess the specificity of the compounds, we compared the RNA-seq profiles of DMSO-, PTC596-, and A1016-treated cells with that of *BMI1*^*KO*^ cells (Figs. 5 and S6). When compared to DMSO, PTC596-treated cells showed more de-regulated genes than A1016-treated cells, many of which being downregulated (Fig. 5a). In contrast, most de-regulated genes in A1016-treated cells were upregulated (Fig. 5a), similarly as observed in BMI1^KO^ cells (Fig. 4d). When directly compared to *BMI1*^*KO*^ cells, PTC596-treated cells presented 4099 significantly de-regulated genes compared to the 2732 de-regulated genes observed in A1016-treated cells (Fig. 5b). Taken together, this suggested significant off-target effects of the PTC596 compound. Using GSEA, we found that genes upregulated in response to *BMI1* knockdown in cancer cells were also upregulated in PTC596 and A1016-treated GBM cells, suggesting efficient drug-mediated BMI1 inhibition (Fig. S6A, B). Treatment with both compounds also succeeded at reducing the expression of glioblastoma proneural genes as observed in *BMI1*^*KO*^ cells (Fig. 5c). In contrast to *BMI1*^*KO*^ or A1016-treated cells however, PTC596 treatment resulted in a molecular signature resembling a mesenchymal phenotype (Fig. S6C), and an enrichment for a gene set involved in EMT (Fig. 5c). Likewise, SOX2-responsive genes were significantly downregulated in PTC596-treated cells, but not in BMI1^KO^ or A1016-treated cells (Fig. 5c). Finally, GSEA revealed a significant up-regulation of EZH2 and PRC2-repressed genes in PTC596-treated cells using three independent gene sets (Figs. 5c and S6D).

**Fig. 6:**
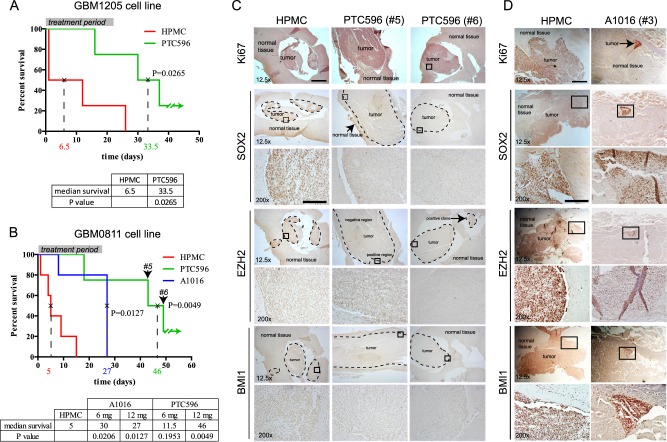
Tumor-bearing mice treated with BMI1 inhibitors show increased lifespan. **a** Kaplan Meier graph for NOD/SCID mice grafted by GBM line 1205 in the brain and treated with BMI1 inhibitors (*n* = 4 for each group) or vehicle (HPMC; *n* = 5). All values are mean ± SEM. **b** Kaplan Meier graph for NOD/SCID mice grafted by GBM line 0811 in the brain and treated with BMI1 inhibitors (*n* = 4 for each group) or vehicle (HPMC; *n* = 5). All values are mean ± SEM. **c** Immuno-histochemistry for BMI1, SOX2, and EZH2 on brain sections from tumor-bearing mice treated or not (HPMC) with PTC596 (GBM0811 cell line). Ki67 was used to confirm cell proliferation in the tumor. Scale bars 12.5×: 1 mm; 200×: 125 μm. **d** Immuno-histochemistry for BMI1, SOX2, and EZH2 on brain sections from tumor-bearing mice treated or not (HPMC) with A1016 (GBM0811 cell line). Ki67 was used to confirm cell proliferation in the tumor. Scale bars 12.5×: 1 mm; 200×: 125 μm.

To evaluate the efficiency of PTC596 at eliminating GBM in vivo, we injected the GBM1205 cell line maintained as neural stem cells in serum-free media in the lateral ventricle of the cerebral cortex of NOD/SCID^IL-2^ mice (*n* = 10 mice). PTC596 was given orally at 12 mg/kg every 3 days for 25 days, and this starting after the first death in the cohort. This protocol was thus designed as an end-stage disease treatment. When compared to HPMC-treated mice (control), PTC596-treated animals showed an extension of median lifespan of 26 days, with one treated animal that was tumor-free 3 months post-treatment (Fig. 6a). Likewise, mice grafted with the GBM0811 cell line (*n* = 16 mice) and treated with HPMC or PTC596 at 6 mg/kg every 3 days also showed an extension of median lifespan by 17 days (Fig. S7). Notably, we could confirm BMI1 and EZH2 down-regulation in the brain tumor during the treatment period and their reactivation 15 days post-treatment in an animal showing relapse (Fig. S8). To simultaneously compare the efficiency of both inhibitors, we injected the GBM0811 cell line in 25 mice. The cohort was composed of 5 HPMC-treated mice, 10 PTC596-treated mice (6 and 12 mg/kg, every 3 days), and 10 A1016-treated mice (6 and 12 mg/kg, daily). When compared to untreated mice, we found that PTC596 and A1016 given at 6 mg/kg extended median survival by 6 and 25 days, respectively (Fig. 6b). At 12 mg/kg, PTC596 extended median lifespan by 41 days, with one tumor-free animal 3 months post-treatment. Mice treated daily with A1016 at 12 mg/kg succumbed from drug toxicity few days after the end of the treatment. Notably, however, all animals treated with A1016, but one, were tumor-free (Fig. 6b). When compared to the tumor of control mice, the tumor of PTC596-treated mice with relapse (mice #5 and #6) had lost many the original characteristics, including reduced BMI1, EZH2, and SOX2 expression (Figs. 6c and S9). In contrast, in the one animal treated with A1016 and that still presented a small tumor of 0.6 mm in diameter, the tumor remained BMI1 and SOX2 positive, although EZH2 levels were reduced (Figs. 6d and S9). These results suggested significant therapeutic effects of both BMI1 inhibitors when used as single agents for the treatment of GBM.

## Discussion

Epigenetic drug therapy may represent a new approach to cure cancers. Herein, we showed that BMI1 inhibitors could efficiently block GBM neural sphere growth and CSC viability in four independent patient-derived GBM cell lines, suggesting that drug-mediated BMI1 inhibition is a versatile treatment for most patients with GBM. Molecular studies revealed that the compounds were able to reduce the BMI1 protein level when used at nanomolar concentration in GBM cell lines having proneural and/or classical phenotypes. When compared to A1016 or *BMI1* knockout, the PTC596 molecule presented additional effects, including inhibition of EZH2, SOX2, and FOXG1. Terminally ill mice bearing brain tumors and treated with PTC596 showed highly extended lifespan. However, analysis of some PTC596-treated animals with relapse revealed that the tumors presented a modified phenotype characterized by reduced BMI1, EZH2, and SOX2 levels.

In a similar strategy to the reprogramming of somatic cells into pluripotent stem cells using a set of four master TFs,^56^ the simultaneous overexpression of *SOX2*, *OLIG2*, *POU3F2*, and *SALL2* reprograms differentiated GBM cells into CSC able to drive tumor formation.^16^ Likewise, glioma-initiating cells can be obtained from tumor suppressor-deficient astrocytes through the ectopic expression of *SOX2*, *OLIG2*, and *ZEB1*.^57^ EZH2 is the catalytic unit of the PRC2 and is overexpressed in GBM. Because EZH2 inactivation impairs cell growth, it prompted interest as a potential target against glioma.^29,46,58,59^ Although EZH2 has a proto-oncogene function, it can also abrogate tumor transformation. Hence, the inactivation of *Ezh2* impairs mouse GBM tumor growth and extends lifespan. Most notably however, prolonged *Ezh2* inactivation causes a loss of the H3K27^me3^ mark, which induces an activation of some pluripotency markers, resulting to a cell fate change and an aggressive tumor transition.^60^ Thus, loss of H3K27^me3^ mark in GBM is predictive for a transition towards a more immature and aggressive phenotype. Notably, Wu et al.^61^ have identified recurrent mutations in *H3F3A* and *HIST3H1B* (encoding for histones H3.3 and H3.1) at positions 27 and 34 (K27M and G34R/V) in about 80% of diffuse intrinsic pontine glioma, a sub-group of pediatric high-grade glioma (pHGG).^61^ Mechanistically, it has been reported that the K27M mutant heterotypic nucleosomes prevents PRC2 binding to the chromatin rather than sequestering the complex.^62,63^ Notably, glioma cells carrying K27M have reduced H3K27^me3^ levels genome-wide leading to an increased expression of developmental genes normally repressed by PRC2.^62,63^

Recently, Jin et al.^40^ proposed dual inhibition of BMI1 and EZH2 as a more efficient therapeutics against glioma than BMI1 (using PTC596) or EZH2 (using EPZ6438) inhibition alone. Drug regimen for PTC596 was once per week at 10 mg/kg. When PTC596 was used in combination with EPZ6438, they observed an increase in median lifespan of 13 days (PN19191 cell line) and of 17 days (Mes20 cell line) when compared to untreated animals, and of 12 and 3 days respectively when compared to PTC596-treated animals.^40^ Considering that the plasma half-life of PTC596 is ~9 h at 10 mg/kg (with an AUC_last_ of ~30 h in the brain), it is predicted that the drug regimen used in these experiments resulted in lack of BMI1 inhibition for several days in between each treatment, allowing BMI1 reactivation in CSCs.

Herein, we found that when used at optimal concentration with a drug regimen allowing relatively constant BMI1 inhibition, PTC596 was able to extend median lifespan by 27 days (GBM1205 cell line) and 41 days (GBM0811 cell line) in terminally ill mice bearing tumors having either a mixed proneural/classical or a proneural phenotype. We found that PTC596 also rapidly impacted on EZH2, SOX2, and FOXG1 protein levels in vitro, possibly resulting in the modified tumor phenotype observed in some animals with relapse. In contrast, the A1016 inhibitor was generally more specific, showing modest effects on EZH2. However, because of its more rapid elimination from the circulation, A1016 was given daily. This resulted in lethal toxicity few days after the end of the treatment when A1016 was used at the highest concentration. Yet, three out four animals were tumor-free at necropsy. We conclude that A1016 would be the best candidate into the clinic for GBM treatment, but that more work is required to control peripheral toxicity in order to achieve high and stable brain concentration during the treatment period.

Interestingly, *BMI1* is overexpressed in cultured neurospheres from childhood brain tumors.^4^ *BMI1* overexpression was also reported in 53% of pHGG in situ and *BMI1* inactivation in pHGG neurospheres impaired tumor formation in mouse xenografts.^64,65^ These findings thus indicate possible additional application for BMI1 inhibitors. A phase 1b study was initiated in 2018 for pHGG using PTC596 in combination with radiation therapy (NCT03605550). Considering that PTC596 can target both BMI1 and EZH2, it is recommended that in vitro and in vivo pre-clinical studies with pHGG cell lines be undertaken first to test for possible de-differentiation toward a more aggressive tumor phenotype. Our findings also raise concern that long-term treatment of solid tumors located outside the central nervous system with PTC596 could drive EMT, thus possibly resulting in a more aggressive and metastatic tumor phenotype.

## Methods

The complete method is available in Supplementary Information. The datasets generated during and/or analyzed during the current study are available from the corresponding author. All blots derived from the same experiment were processed in parallel. Primary GBM were obtained from the Department of Pathology of the Maisonneuve-Rosemont Hospital, and the Brain Tumor Tissue Bank (Toronto, ON, Canada). Fresh tumors were obtained from consenting patients and used with permission of our institutes’ ethical committee (CER; Project No. 2007-402, 06099). The Maisonneuve-Rosemont Hospital’s animal protection committee (CPA) approved all experiments performed in mice (Project No. 2015-23). Fresh GBM samples were processed for cell cultures within 1 h after reception. Tumor was washed and cut in small pieces before mechanical dissociation in oxygenated HBSS. Cell suspensions were passed over a 40-μm filter mesh. After centrifugation, cells were resuspended in GBM media: DMEM/F12 medium (Invitrogen) containing 0.25% glucose, N2 and B27 supplements, Heparin (2 μg/ml; Sigma), gentamicin (25 μg/ml; Invitrogen), human recombinant FGF2 (10 ng/ml; Peprotech), and human recombinant EGF (20 ng/ml; Sigma). Afterward, cultures were allowed to grow for 3 weeks to form spherical colonies (i.e., neurospheres). For passages, neurospheres were incubated in an enzyme-free solution (Millipore Bioscience Research Reagents) at 37 °C for 5 min, and mechanically dissociated with a 20G needle. After trituration, the cell suspension was plated in GBM media. Cell lines used in this study are: GBM1205, GBM1909, GBM0811, and GBM0410. Molecular weights for western blot analysis are showed with the original blots in Supplementary Information as Supplementary Figs. 10 and 11. BMI1 inactivation using CRISPR/Cas9 were carried out by the polymeric delivery of a Cas9-expressing plasmid (Dharmacon #CAS10140), a synthetic guide RNA (sgRNA) Scramble (Dharmacon #U-007501) or complementary to BMI1 (Target: AACGTGTATTGTTCGTTACC) and a synthetic trans-activating crRNA (Dharmacon #U-002005) using Mirus TransIT-X2 (Cat#MIR6003) according to the manufacturer's instructions. To enhance the knockout efficiency, a full six-well plate of neurospheres was dissociated and condensed in 1 ml of GBM media and platted onto a well of an ultra-low attachment six-well plate.

### Reporting Summary

Further information on research design is available in the Nature Research Reporting Summary linked to this article.

## Supplementary information

Supplementary Information

Reporting Summary

## Data Availability

Raw data, cell lines, and reagents are available upon request.

## Code availability

Accession code for RNA-seq data presented in Figs. 4 and 5 is GSE136829.
