# Supplementary material for: Off-target effect of the BMI1 inhibitor PTC596 drives epithelial-mesenchymal transition in glioblastoma multiforme
Source: NPJ Precis Oncol. 2020 Jan 6;4:1. doi: 10.1038/s41698-019-0106-1 (PMC6944693; doi:10.1038/s41698-019-0106-1)
Supplement: Supplementary file 1 — Supplementary Information [file 41698_2019_106_MOESM1_ESM.pdf]

SUPPLEMENTARY INFORMATION

Off-target effect of the BMI1 inhibitor PTC596 drives epithelial-mesenchymal transition in glioblastoma multiforme

Anthony Flamier[1†], Mohamed Abdouh[1†], Rimi Hamam[1], Andrea Barabino[1], Niraj Patel[1], Andy Gao[1], Roy Hanna[1] and Gilbert Bernier[1, 2*]

[1] Stem Cell and Developmental Biology Laboratory, Hôpital Maisonneuve-Rosemont, 5415 Boul. l'Assomption, Montréal, Canada, H1T 2M4

[2] Department of Neurosciences, University of Montreal, Montreal, Canada

[†]These authors contributed equally to this work

*Corresponding author

Tel: 514-252-3400 ext. 4648

E-mail: gbernier.hmr@ssss.gouv.qc.ca

Content:

Supplementary Materials and Methods

Supplementary Figure Legends

11 Supplementary Figures

SUPPLEMENTARY INFORMATION

SUPPLEMENTARY METHODS

Differentiation of human pluripotent stem cells

The differentiation protocol was based on a previous study (Espuny-Camacho et al., 2013). However, the Noggin agonist LDN193189 was used to reduce recombinant Noggin concentration. Human embryonic or induced pluripotent stem cells were dissociated using Accutase (Innovative Cell Technology #AT-104) and platted on growth factor reduced matrigel (Corning #356231) in mTeSR1 media (Stem Cell Technologies) supplemented with ROCK inhibitor (Y-27632;10μM, Cayman Chemical #10005583). Upon 70% of confluency, the media was changed to DDM supplemented with B27 (1X final), Noggin (10 ng/ml, PeproTech #120-10C) and LDN193189 (0.5μM; Sigma #SML0559). The medium was changed every day. After 16 days of differentiation, the medium was changed to DDM/B27 and replenished every day. At day 24, neural progenitors were manually detached from the plate and subsequently mixed in DDM/B27 supplemented with ROCK inhibitor (Y-27632; 10μM) and platted on growth factor reduced matrigel coated plates or chamber slides (LabTek #154534). Five days after the dissociation, half of the medium was changed for Neurobasal A media supplemented with B27 (1X final) and changed again every three days for a total of 30 days.

Immunofluorescence microscopy

All secondary antibodies were tested alone or in combination to assay for possible non-specific background fluorescence. Cells were fixed with 4% PFA for 15 min and permeabilized with Triton X-100 for 10 min. Unspecific antigen blocking was performed using 1% BSA in PBST for 30 min. Cells were incubated with the primary antibody overnight at 4C in a humidified chamber. After incubation with the secondary antibody, slides were counter stained with DAPI. Pictures were taken using a confocal microscopy system (Olympus). For figure 2E-F, GBM neurospheres were fixed overnight with 4% PFA, dehydrated using sucrose gradient baths and cryo-sectioned (20μm) prior to permeabilization.

SUPPLEMENTARY INFORMATION

List of antibodies :

Name<br>Reactivity<br>(h:Human; ms:Mouse)	Source	Cat#	Dilution IF/IHC	Dilution WB
**BMI1 (D42B3)**	Cell Signaling	5856		1/500
**SOX2**	Cell Signaling	9661	1/400	1/1000
**CD133**	Chemicon	MAB4310		1/500
**FOXG1**	Abcam	ab18259	1/400	1/1000
**EZH2**	CST	3147S	1/400	1/1000
**GAPDH (D-6)**	SCB	sc-166545		1/250
**H2Aub (D27C4)**	Cell Signaling	8240	1/1600	1/2000
**Histone H3 (C-16)**	SCB	sc-8654		1/250
**MAP2**	Abcam	ab5392	1/10000	
**Nestin (C-20)**	SCB	sc-21247	1/50	1/250
**GFAP**	Dako	Z0334	1/400	

Western Blot

Cell extracts were homogenized in the Complete Mini Protease inhibitor cocktail solution (Roche Diagnostics), followed by sonication. Protein material was quantified using the Bradford reagent. Proteins were resolved in 1x Laemlli reducing buffer by SDS-PAGE electrophoresis and transferred to a Nitrocellulose blotting membrane (Bio-Rad). Subsequently, membranes were blocked for 1h in 5% non-fat milk-1X TBS solution and incubated overnight with primary antibodies. Membranes were then washed 3 times in 1X TBS; 0.05% Tween solution and incubated for 1h with corresponding horseradish peroxidase-conjugated secondary antibodies. Membranes were developed using the Immobilon Western (Millipore). Blots were quantified using the Image quant program.

SUPPLEMENTARY INFORMATION

Real-time RT-PCR

RNA was isolated using TRIzol reagent (Invitrogen). Reverse transcription (RT) was performed using 1 µg of total RNA and the MML-V reverse transcriptase (Invitrogen). Real-time PCR was carried in triplicates using Platinum SYBRGreen Supermix (Invitrogen) and Real-time PCR apparatus (ABI prism 7002). Primers used were:

Gene	Forward	Reverse
Gene expression		
**GAPDH**	TCACCAGGGCTGCTTTTAAC	ATCCACAGTCTTCTGGGTGG
**BMI1**	TCATCCTTCTGCTGATGCTG	CCGATCCAATCTGTTCTGGT
**CD133**	AGTCGGAAACTGGCAGATAGC	GGTAGTGTTGTACTGGGCCAAT
**EZH2**	AATCAGAGTACATGCGACTGAGA	GCTGTATCCTTCGCTGTTTCC
**ZFHX4**	ACAGACTGGGGCAAATAGCAA	GGTAGAACGACATAGGTGCAGAA
**NES**	CTGCTACCCTTGAGACACCTG	GGGCTCTGATCTCTGCATCTAC

RNA sequencing

GBM line 120516 was used for all RNA sequencing analyses. GBM cells treated with a non-template gRNA, a gBMI1, DMSO, PTC596 (50nM) or A1016 (50nM) for 24 hours were collected and validated by immunoblotting for BMI1 reduction. Once validated RNA were extracted using RNeasy kit (Qiagen) according to manufacturer instructions including DNase treatment. RNA integrity was controlled using Bioanalyzer (Agilent) and two samples from each group with a RIN>8 were sequenced. 1 □g of total RNA was subjected to mRNA isolation using OligodT Dynabeads and libraries were prepared using Ion Total RNA-Seq Kit v2. Libraries were sequenced onto P1 chips from Ion torrent as unpaired to reach 40 millions of reads for each sample.

RNA sequencing analyses

Raw sequencing files (FASTQ) were first trimmed to remove adapters and further validated using FASTQC v0.11.7. Reads were aligned onto hg19 using Hisat2 v2.1.0 with default parameters and sorted using samtools v1.9. Gene level counts were determined using featureCounts from subread package. Differential expression analyses were performed using DEseq2 package in R and volcano plot were generated using Tmisc and Calibrate R packages. FPKM values for each sample were calculated using Cufflinks v2.2.1. Heatmaps were generated with FPKM values using

Heatmapper2. Z-scores were computed for each row and clustered using average linkage and Pearson distance measurement method. For Gene-Set Enrichment Analysis (GSEA), differentially expressed genes were pre-ranked according to the log2 of fold change and a p-value < 0.05. Ranked list were then subjected to GSEA with a classic scoring scheme, an enrichment score normalization and 1000 permutations. Publicly available RNA-seq data were obtained from the Ivy Glioblastoma Atlas Project and compared to our data sets.

Intracranial cell transplantation experiments

Animals were handled in strict accordance with the Animal Care Committee of the Maisonneuve-Rosemont Hospital Research Centre. Cells were resuspended in oxygenated HBSS and 3□l aliquots were injected stereotactically into 60-d-old nonobese diabetic/severe combined immunodeficiency (NOD/SCID) mouse brain, after administra- tion of general anesthesia (Somnotol; 60 mg/kg). Mice were placed in the stereotactic frame using ear bars and a hole was bored in the skull. The injection coordinates were 2 mm to the right of the midline, 2 mm posterior to the coronal suture and 3 mm deep. The scalp was closed with wound clips (Harvard Apparatus). Animals were followed daily for development of neurological deficits. For quanrtification of IHC, the intensity from ten random regions of equal size was measured for each sample using Fiji. The background intensity of each sample was then subtracted from the corresponding measures. Expression levels in treated tumor samples were normalized over the control samples.

Statistical analysis

Statistical analysis was performed using Graphpad software (Prism 6). Statistical differences were analyzed using Student's *t*-test for unpaired samples. In all cases, the criterion for significance (*P* value) was set as mentioned in the figures. When comparisons were made using independent samples of equal size and variance following a normal distribution, significance was assessed using an unpaired two-sided Student's t-test. Where several groups were compared, significance was assessed by ANOVA and adjusted for multiple comparisons using the Bonferroni correction.

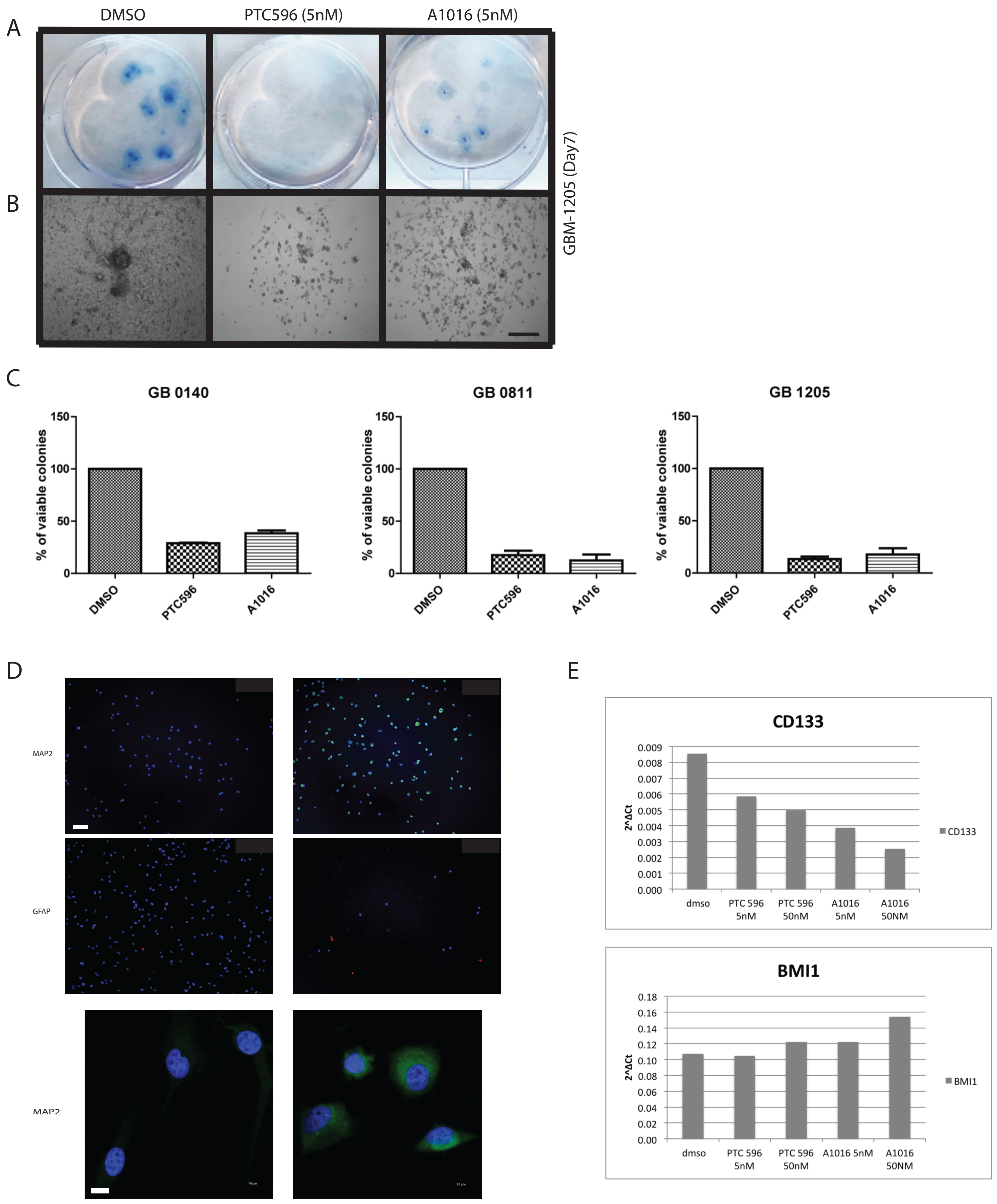

Figure S1. BMI1 inhibitors induce cell death and growth retardation in GBM cells
(A) Colony forming assay on adherent culture conditions (Matrigel) after 7 days of treatment with DMSO, PTC596 (5nM) or A1016 (5nM). Left: Cresyl violet staining showing the growth of colonies. Right: higher magnification of adherent cells. Note the presence of apoptotic cells in PTC596 and A1016 treatments.
(B) Higher magnification of A. Scale bar: 50μm
(C) Quantification of viable colonies from (A-B) for 3 independent GBM cell lines.
(D) Immunofluorescence for the dendritic marker MAP2 and the glial marker GFAP on GBM cell line 1909 in adherent condition treated for DMSO or PTC596. Scale bars: 100μm (top); 10μm (bottom).
(E) Gene expression levels by qRT-PCR for cancer stem cell markers BMI1 and CD133 (PROM1) in GBM line 1205 after treatment with DMSO or increasing concentrations of BMI1 inhibitors.

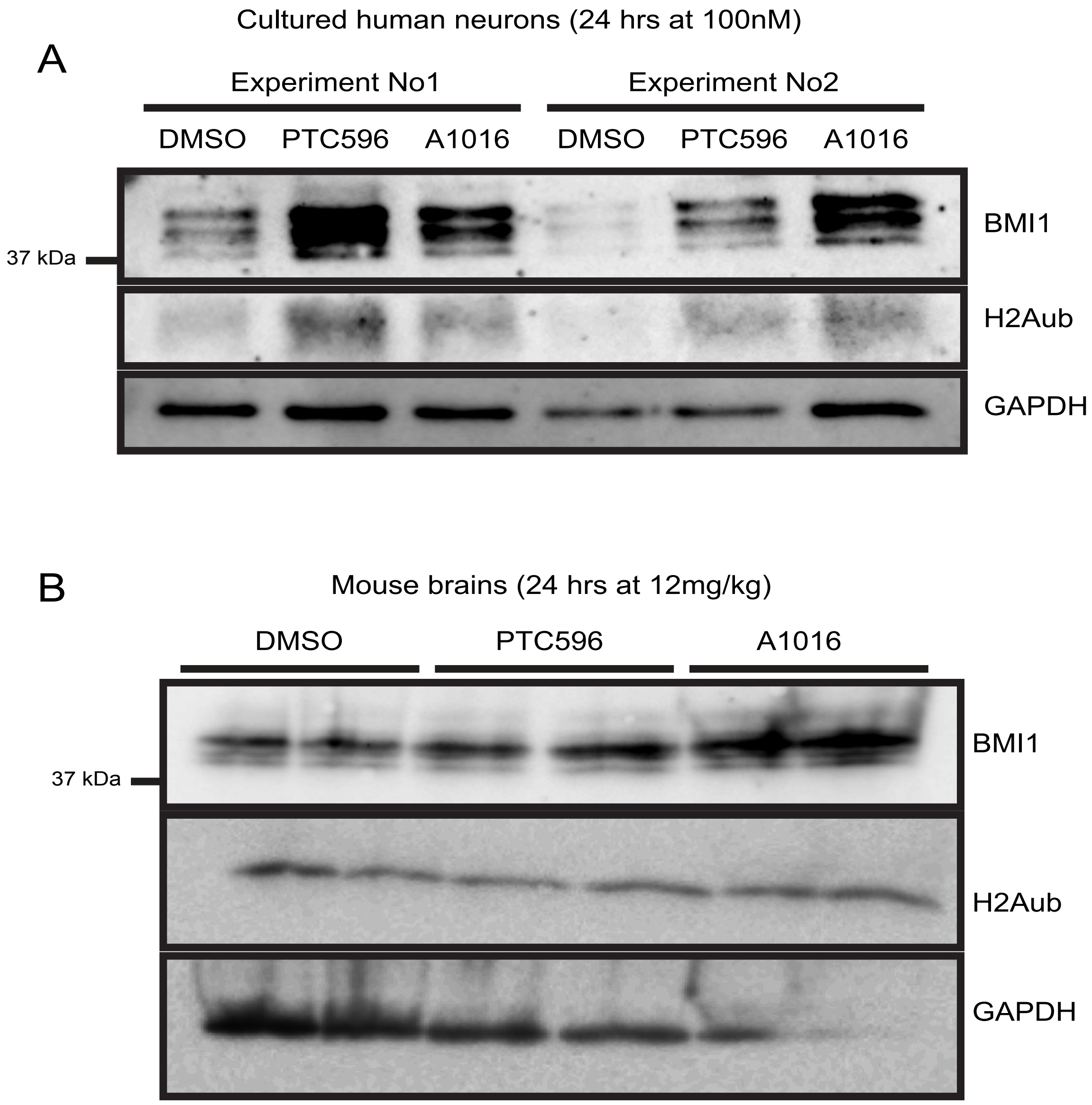

Figure S2. BMI1 inhibitors induce BMI1 accumulation in post-mitotic neurons
(A) iPSC-derived human neurons at day in vitro 30 were exposed to DMSO (diluent), PTC596 or A1016 for 24h. Cellular extracts were then analyzed by Western blot, revealing accumulation of BMI1 in PTC596 and A1016-treated cells. Likewise, H2Aub was also increased in drug-treated samples.
(B) Wild type adult (P45) mice were given 12mg/kg of PTC596 or A1016 through oral administration, and mouse were brains analyzed 24h after. Note that A1016 exposure apparently resulted in elevated Bmi1 levels.

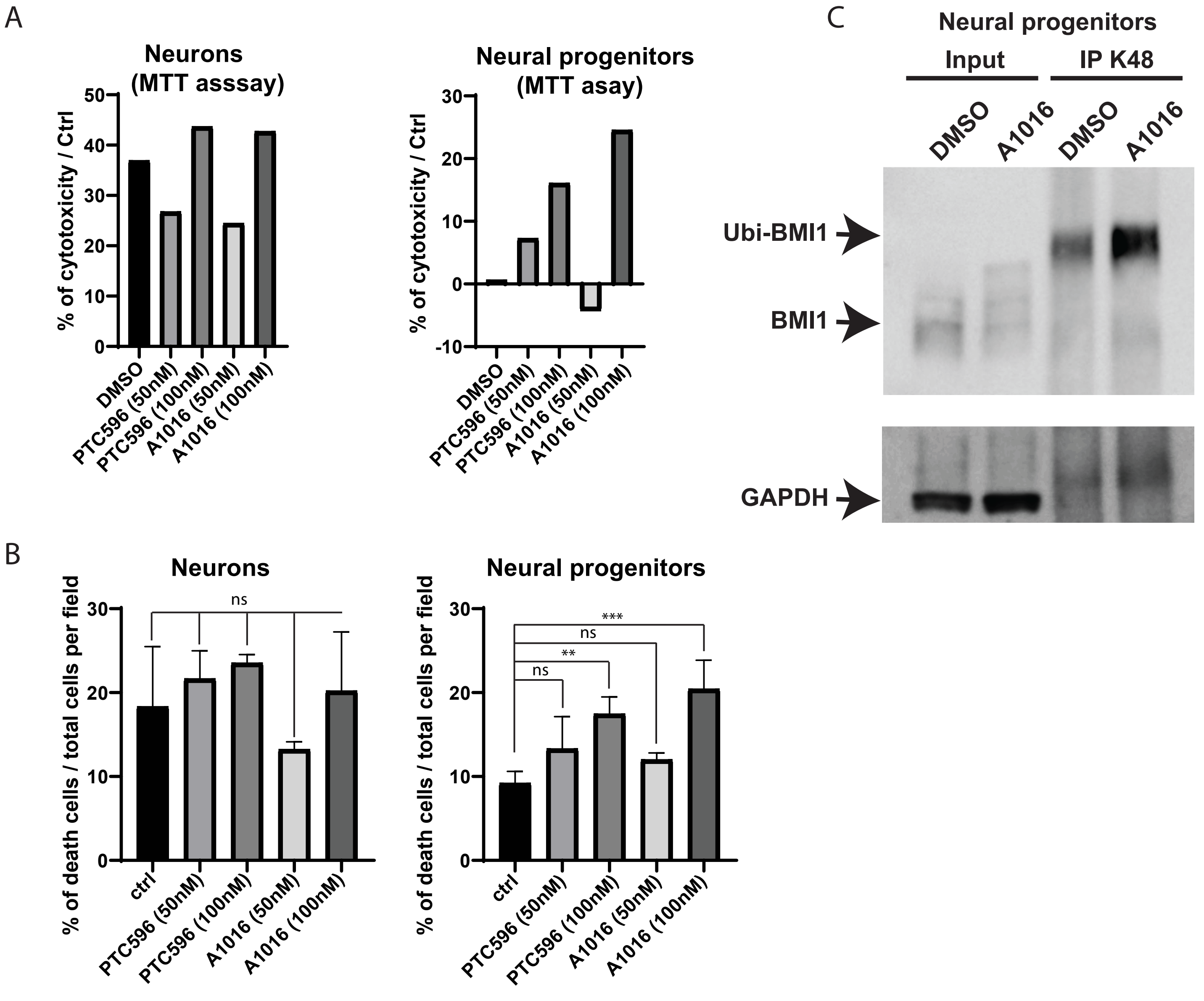

Figure S3. BMI1 inhibitors are minimally toxic for in post-mitotic human neurons
(A-B) iPSC-derived human neurons at day in vitro 30 and iPSC-derived neural progenitors at day in vitro 15 were exposed to DMSO (diluent), PTC596 or A1016 for 24h. Cellular extracts were then analyzed using the MTT assay or by measuring the number of death cells over the total number of cells. This revealed that PTC596 and A1016 were minimally toxic for neurons but presented dose-dependent toxicity for neural progenitors.
(C) iPSC-derived neural progenitors at day in vitro 15 were exposed to DMSO (diluent) or A1016 for 24h. Native cell extracts were immune-precipitated with an antibody against poly-ubiquitinated proteins at lysine 48 (K48), a mark for protein degradation by the proteasome. Western blot analysis of the samples revealed that BMI1 levels were reduced upon A1016-exposure (input), and that the total amount of poly-ubiquitinated BMI1 K48 was increased upon A1016-exposure.
All values are mean +/- SEM. (**) P-Value <0,01; (***) P-Value <0,001.

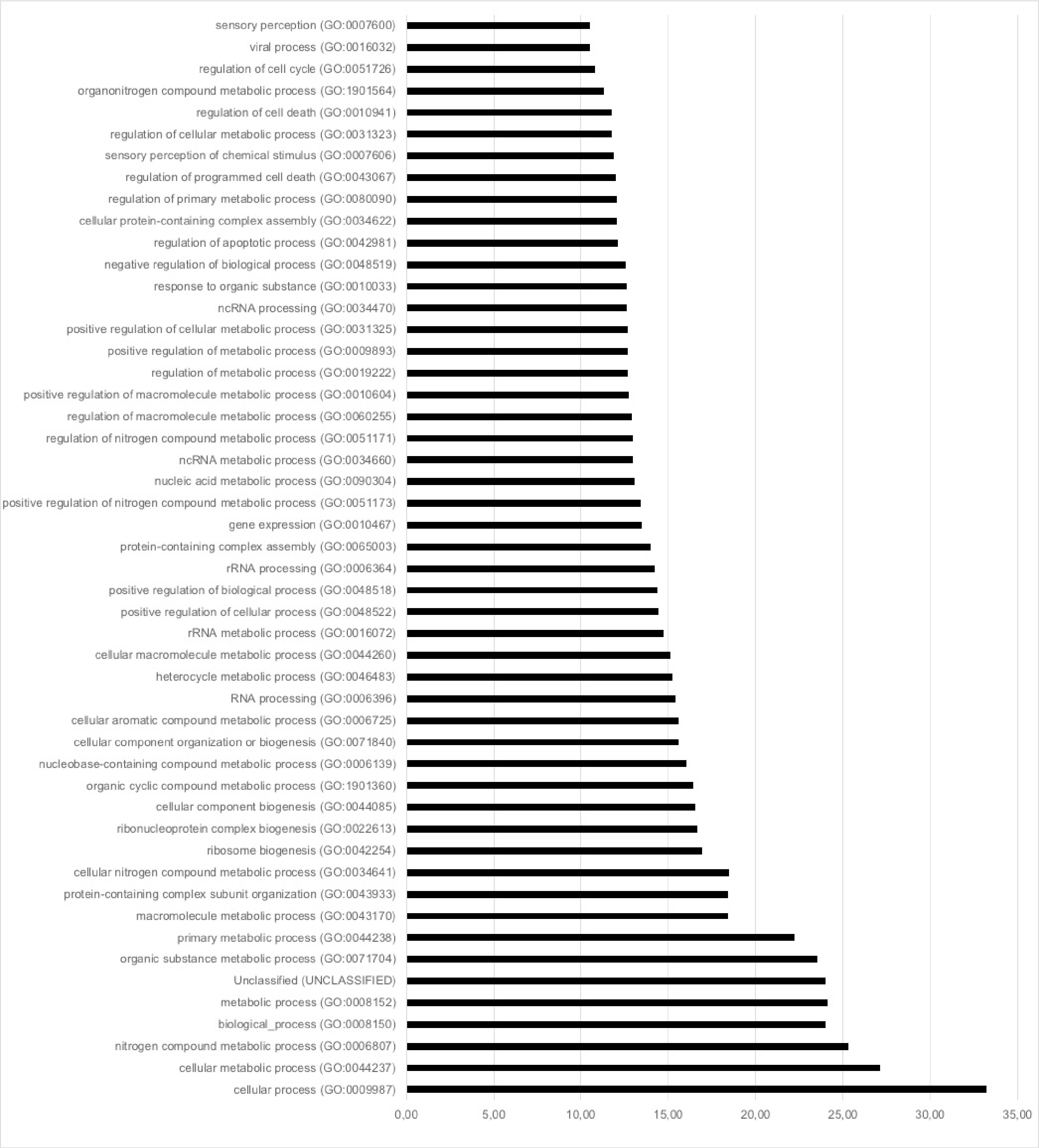

Figure S4. Gene ontology annotation of the most up-regulated genes in BMI1KO GBM cells Note the up-regulation of several pathways associated with metabolic processes.

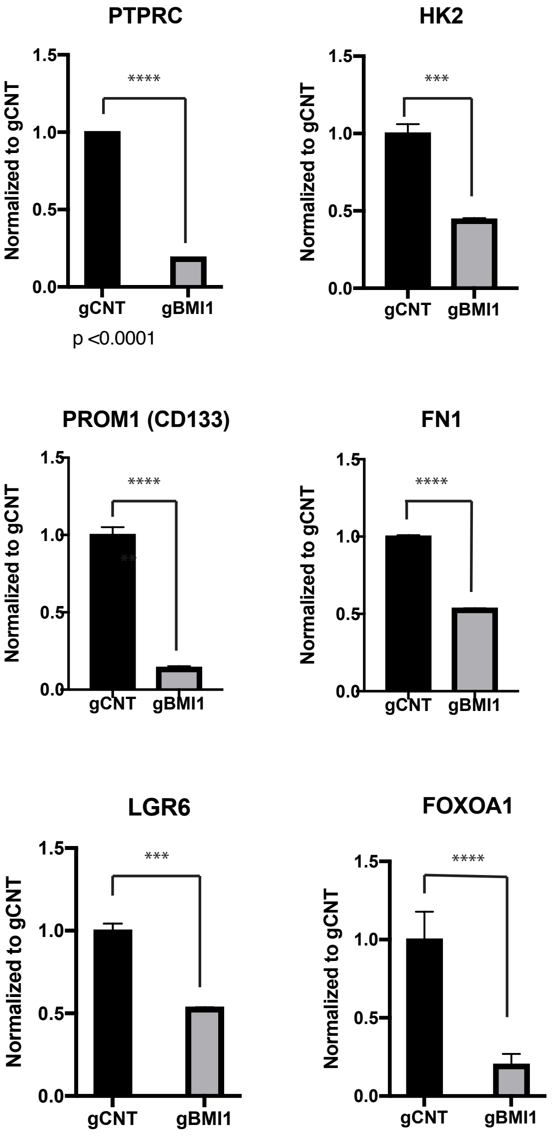

Figure S5. Expression levels of stem cell genes down-regulated in BMI1KO GBM cells Confirmation that most down-regulated stem cell genes detected in BMI1KO GBM cells using RNA-seq are truly down-regulated as determined using real-time RT-PCR analysis.

All values are mean +/- SEM. (***) P-Value <0,001; (****) P-Value <0,0001.

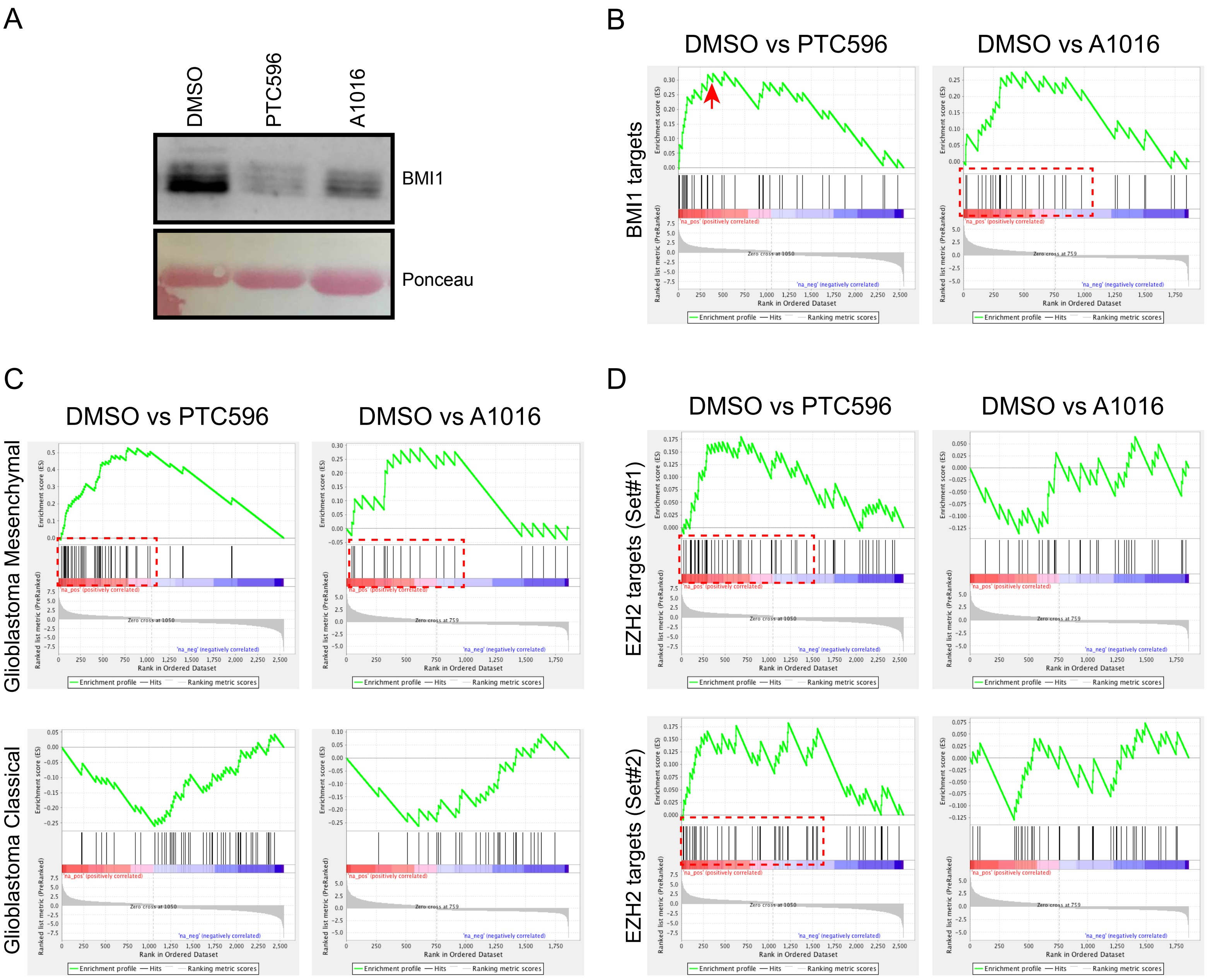

Figure S6. Enrichment for mesenchymal and EZH2 target genes in PTC596 treated GBM cells.
(A) Immunoblot of BMI1 in GBM line 120516 treated for 24 hours with DMSO, PTC596 or A1016. These samples were used for RNA-seq analyses. Ponceau was used as loading control.
(B) Gene-Set Enrichment Analysis (GSEA) showing enrichment for a gene set upregulated upon drug-mediated BMI1 inhibition.
(C) Gene-Set Enrichment Analysis (GSEA) showing enrichment for gene sets upregulated in Mesenchymal or Classical glioblastoma.
(D) Gene-Set Enrichment Analysis (GSEA) showing enrichment for gene sets containing EZH2 targets. (B-D) Red arrows and boxes highlight enrichments of interest.

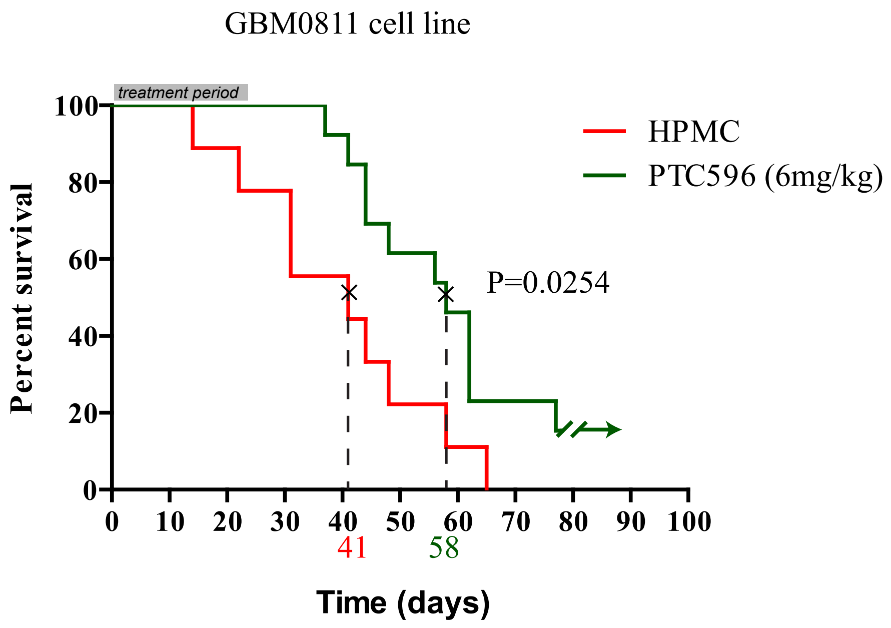

Figure S7. PTC596 treatment at 6mg/kg improves lifespan in brain tumor-bearing mice
Kaplan Meier graph for NOD/SCID mice grafted by GBM line 0811 in the brain and treated with BMI1 inhibitors (n=8 for each group) or vehicle (HPMC; n=8).

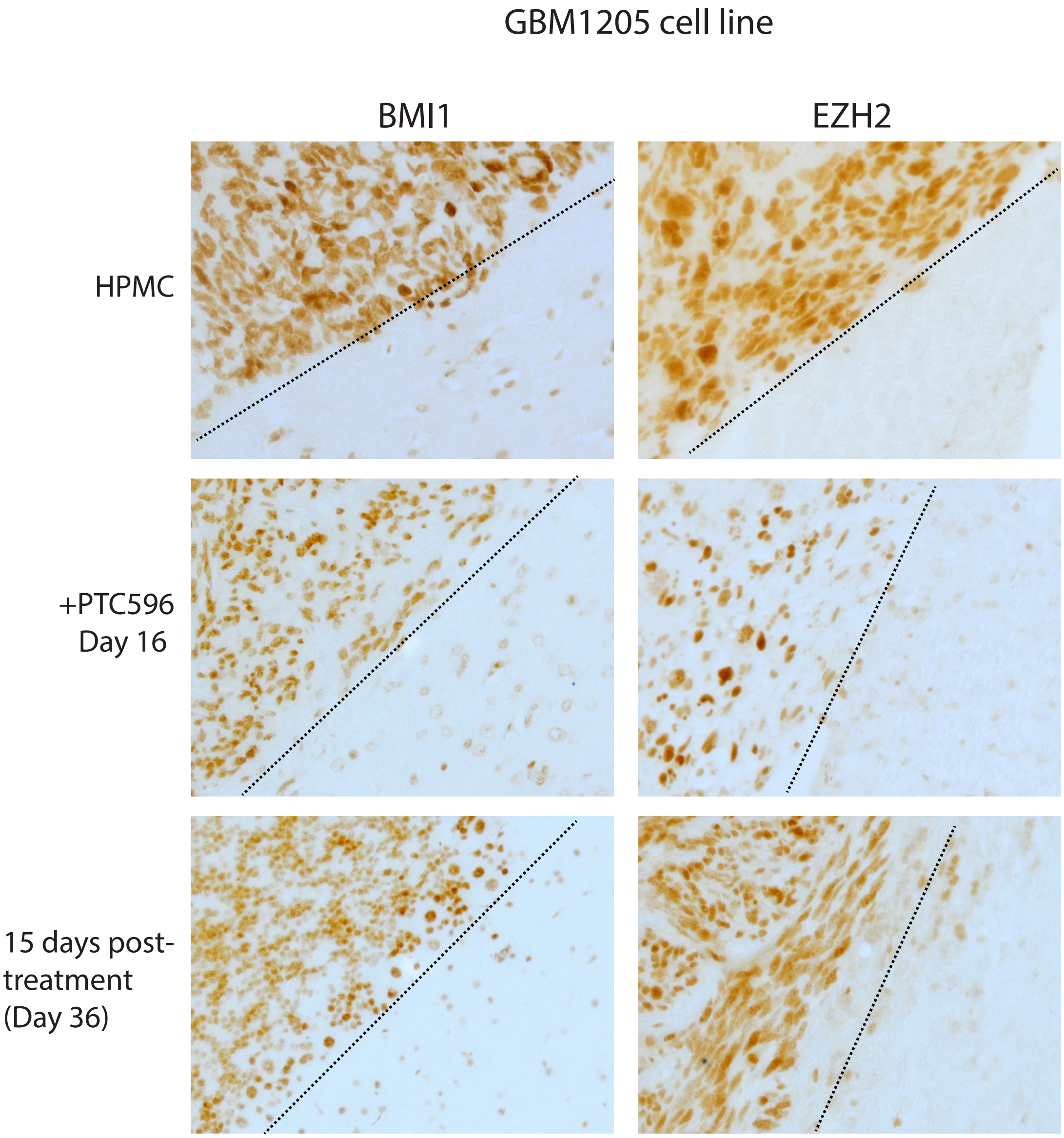

Figure S8. Acute PTC596 treatment reduces BMI1 and EZH2 expression in brain tumor-bearing mice IHC of human brain tumors from untreated (HPMC), PTC596-treated (+PTC596) and 15 days post-treatment mice. Note reduced BMI1 and EZH2 levels in PTC596-treated mice at day 16.

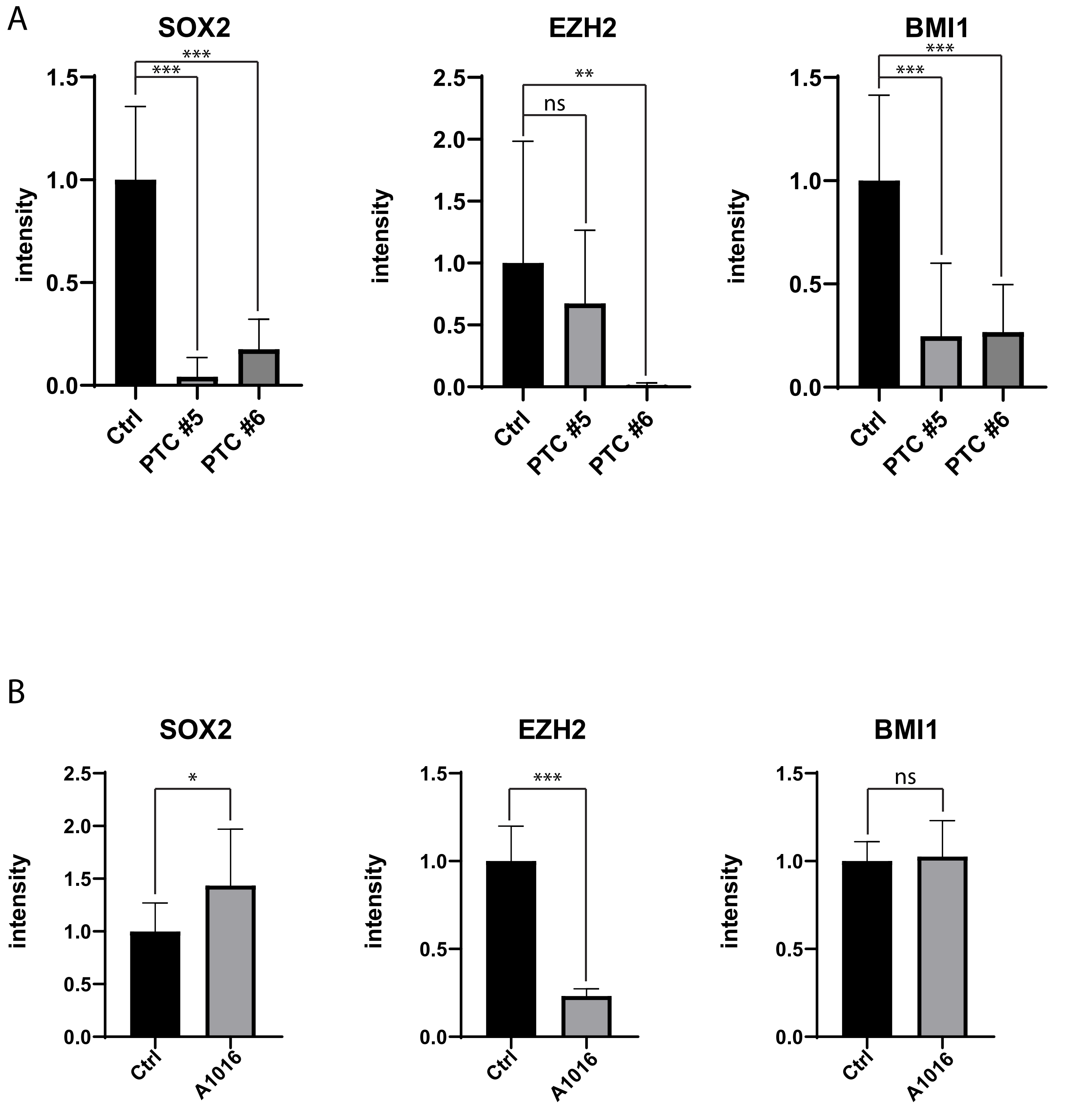

Figure S9. Relapse in PTC596-treated mice display an altered tumor phenotype
IHC of human brain tumors (see Figure 6 as reference) from untreated, PTC596-relapse or A1016-treated mice were quantified. This revealed that the expression of SOX2, EZH2 and BMI1 were significantly reduced in relapse from PTC596-treated mice. In contrast, only EZH2 expression was affected in the only tumor observed in A1016-treated mice.
All values are mean +/- SEM. (*) P-Value <0,05; (**) P-Value <0,01; (***) P-Value <0,001; (****) P-Value <0,0001.

Figure 2A

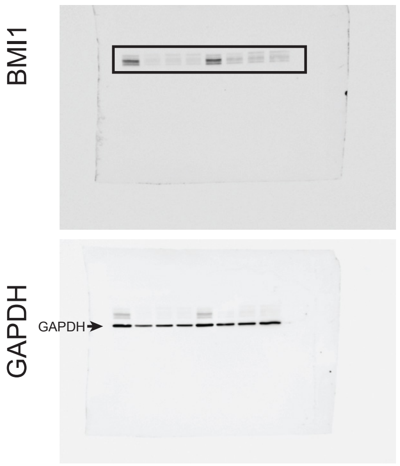

Figure 2B

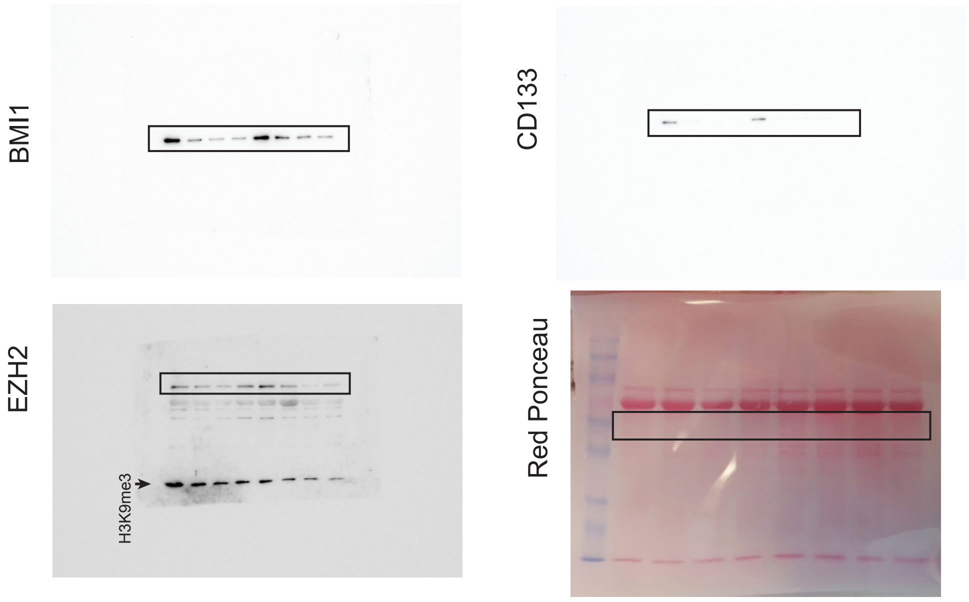

Figure 2C

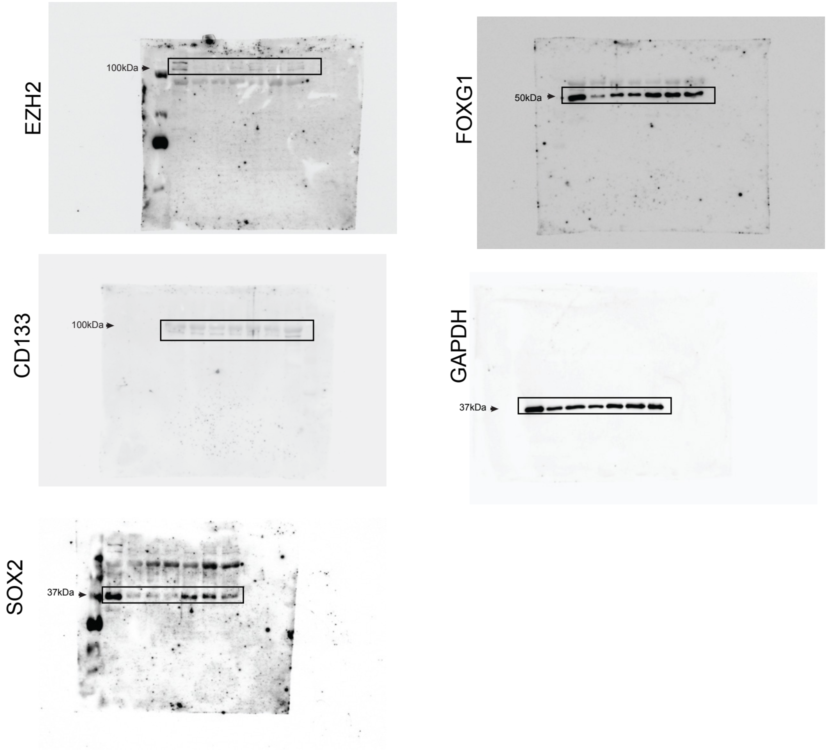

Figure 3A

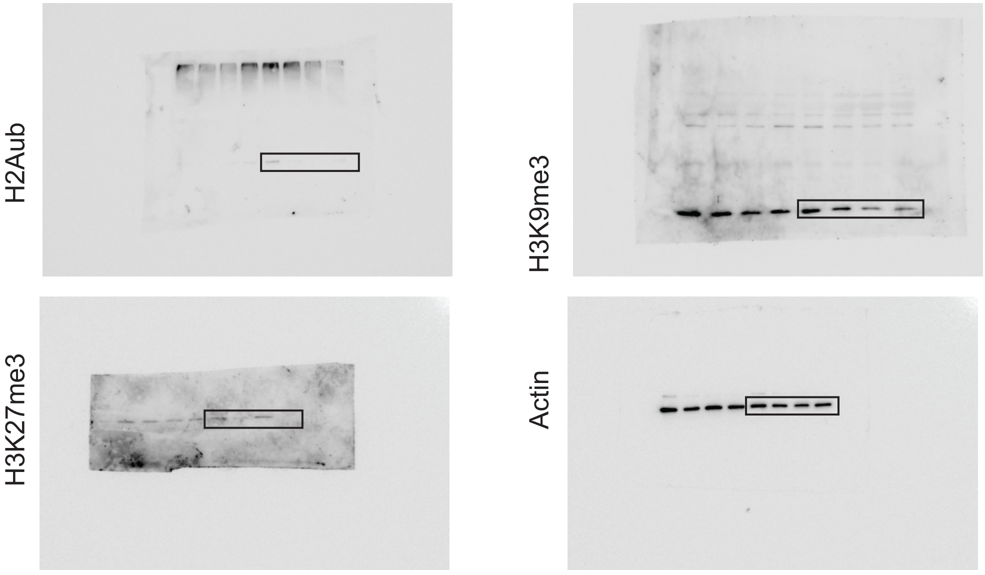

Figure 4C

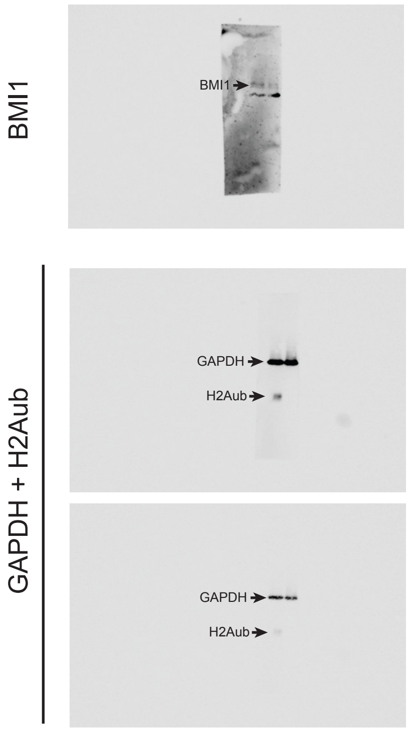

Figure S10. Original blots from the manuscript
Original immuno-blots from the main manuscript.

Figure 2A

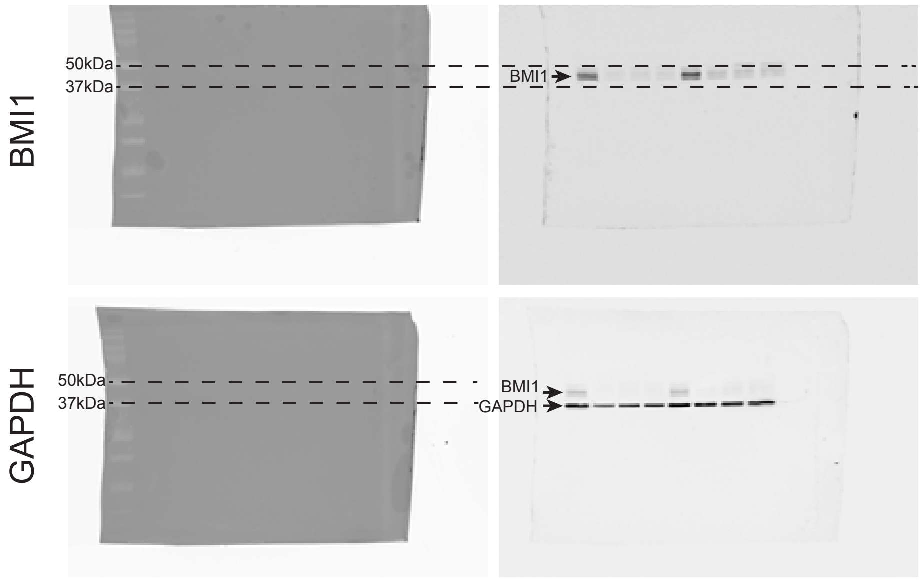

Figure 4C

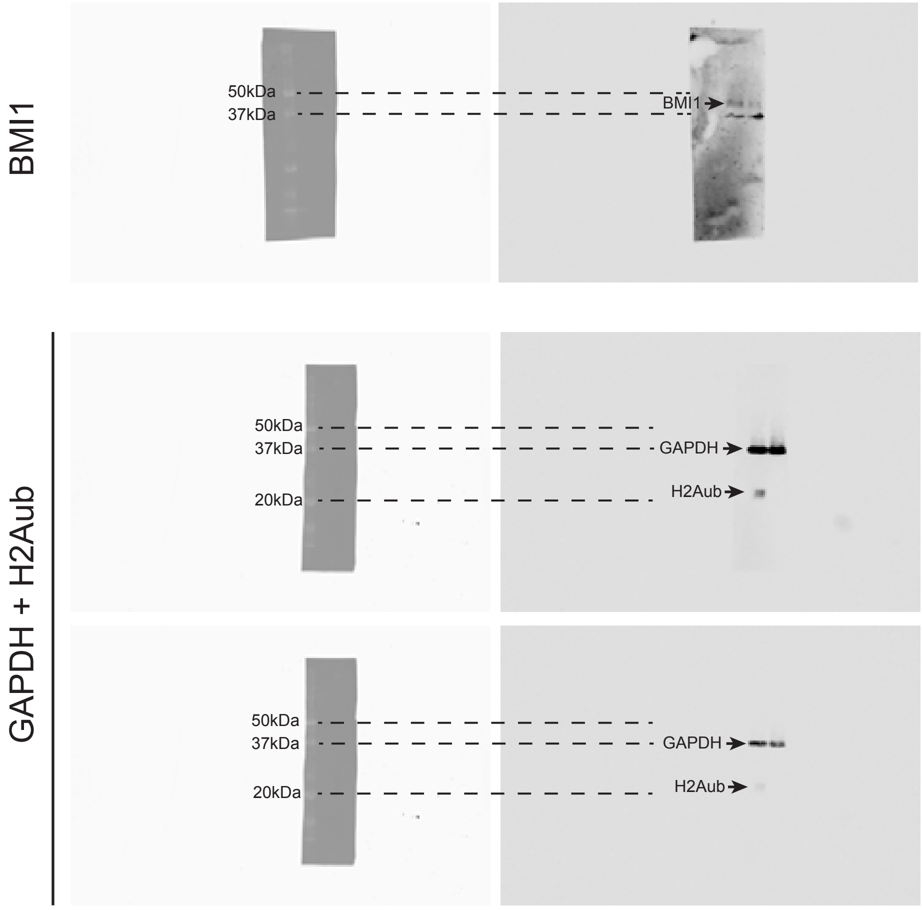

Figure S11. Original blots from the manuscript with molecular weight
Most important immuno-blots from the main manuscript with the indicated molecular weight.
